# MyD88 determines the protective effects of fish oil and perilla oil against metabolic disorders and inflammation in adipose tissue from mice fed a high-fat diet

**DOI:** 10.1038/s41387-021-00159-y

**Published:** 2021-06-17

**Authors:** Feng Wang, Mingyuan Hu, Hangju Zhu, Chao Yang, Hui Xia, Xian Yang, Ligang Yang, Guiju Sun

**Affiliations:** 1 Key Laboratory of Environmental Medicine and Engineering of Ministry of Education, and Department of Nutrition and Food Hygiene, School of Public Health, Southeast University, Nanjing, China; 2 Tianjin Institute of Environmental and Operational Medicine, Tianjin, China; 3 Wannan Medical College, Wuhu, China; 4 Jiangsu Cancer Hospital, Nanjing, China

**Keywords:** Obesity, Cardiovascular diseases

## Abstract

**Background:**

The beneficial effects of ω−3 polyunsaturated fatty acids (PUFA) vary between different sources. However, there is a paucity of comparative studies regarding the effects and mechanisms of marine and plant ω−3 PUFA on obesity.

**Objective:**

The aim of this study was to evaluate the effects of fish oil (FO) and perilla oil (PO) on glucolipid metabolism, inflammation, and adipokine in mice fed a high-fat (HF) diet in association with the contribution of toll-like receptor 4 (TLR4)/myeloid differentiation primary response 88 (MyD88) pathway.

**Methods:**

C57BL/6J mice and MyD88−/− mice were randomly divided into 4 groups: normal chow diet, HF diet, HF diet accompanied by daily gavage with either FO or PO. After 4 weeks, blood biochemistries, adipocyte histology, mRNA, and protein expression of MyD88-dependent and -independent pathways of TLR4 signaling in epididymal adipose tissue were measured.

**Results:**

In C57BL/6J mice, there were no statistical differences between FO and PO in decreasing body weight, glucose, insulin, triglyceride, total cholesterol, interleukin-6, and increasing adipocyte counts. FO and PO decreased mRNA and protein expression of TLR4, MyD88, tumor necrosis factor receptor-associated factor 6, inhibitor of nuclear factor kappa B kinase beta and nuclear factor-kappa B p65. In MyD88−/− mice, the beneficial effects of FO and PO on HF diet-induced metabolism abnormalities and inflammation were abolished. FO and PO had no impacts on mRNA and protein expression of receptor-interacting protein-1, interferon regulate factor 3, and nuclear factor-kappa B p65.

**Conclusion:**

FO and PO exhibit similar protective effects on metabolic disorders and inflammation through inhibiting TLR4 signaling in a manner dependent on MyD88. These findings highlight plant ω−3 PUFA as an attractive alternative source of marine ω−3 PUFA and reveal a mechanistic insight for preventive benefits of ω−3 PUFA in obesity and related metabolic diseases.

## Introduction

Obesity is one of the major public health issues in the world. It causes excess fat in adipose tissue, leading to metabolic disorders, which are mainly characterized by dyslipidemia and insulin resistance. ω−3 polyunsaturated fatty acids (PUFA) have been verified to benefit vascular health. A recent science advisory from American Heart Association addressed the use of ω−3 PUFA to reduce triglyceride (TG) in patients with hypertriglyceridemia, either as monotherapy or as an adjunct to other lipid-lowering agents^1^. In addition, American Diabetes Association recommended eating foods rich in ω−3 PUFA for type 2 diabetes mellitus^2^.

ω−3 PUFA can be classified into marine and plant types. The former are mainly eicosapentaenoic acid (EPA) and docosahexaenoic acid (DHA) enriched in fish oil (FO), krill oil, and fish. The latter is α-linolenic acid (ALA), which is abundant in perilla oil (PO), flaxseed oil, and camelina sativa oil. Dietary ω−3 PUFA sources are of pivotal importance for metabolic risk. For example, intake of marine, but not plant, ω−3 PUFA was associated with low cardiovascular mortality among patients with type 2 diabetes^3^. FO and krill oil, even if they are all rich in marine ω−3 PUFA, had different postprandial plasma lipidomic responses in diacyl-phospholipids and ether-phospholipids^4^. In our previous work, we found that FO improved TG and high-density lipoprotein-cholesterol (HDL-C) in type 2 diabetic patients with abdominal obesity^5^. We also indicated that PO alleviated hypertriglyceridemia in KKAy mice^6^. However, whether administration of PO is able to mimic the effects of FO remains obscure.

ω−3 PUFA are major ligands of peroxisome proliferator-activated receptors. Their binding induced transcription of specific genes encoding inflammation in adipose tissue^7^. The health benefits of ω−3 PUFA were associated with suppression of toll-like receptor 4 (TLR4)^8,9^. TLR4, highly expressed in adipocytes, is acknowledged as one of the main triggers of the obesity-induced inflammatory signaling pathways. C57BL/6J mice lacking TLR4 had protective effect against high-fat (HF) diet-induced insulin resistance, possibly due to reduced adipose tissue inflammation^10^. Stimulation of TLR4 facilitated the myeloid differentiation 88 (MyD88)-dependent and -independent pathways, resulting in generation of pro-inflammatory cytokines^11^, but little work has been done on which pathway determines the protective effects of ω−3 PUFA.

According to these considerations, the purpose of this study was to evaluate the effects of FO and PO on glucolipid metabolism, inflammation, and adipokine in association with the contribution of MyD88-dependent and -independent pathway of TLR4 signaling.

## Materials and methods

### Animals and treatments

Six-week-old male C57BL/6J mice and 8-week-old male MyD88−/− mice with a C57BL/6J background were obtained from Model Animal Research Center of Nanjing University (Nanjing, China). Mice were housed in a controlled environment (22 ± 2 °C, 40%–60% humidity, and 12-h light/12-h dark cycle) with free access to water and food. After 1 week of acclimatization, mice were randomly divided into 4 groups (*n* = 10 C57BL/6J, *n* = 6 MyD88−/−, for each group): normal chow (NC) diet (AIN-93M, 9.5 kcal% from fat, formulated by American Institute of Nutrition), HF diet (D12451, 45.4 kcal% from fat, formulated by Research Diets, Inc.), HF diet accompanied by daily gavage with either 0.5 g/kg bw FO or 0.5 g/kg bw PO. The sample size was determined in accordance with references^12–15^. The fatty acid profile of the diets and oils is shown in Tables S1–S3. The concentration of FO and PO was established by our previous report^16^. No blinding was done. After 4 weeks, serum and epididymal adipose tissue were collected for further analysis. All animal experimental procedures were approved by the Institutional Animal Care and Use Committee at Southeast University.

### Biochemical measurement

Serum glucose, TG, total cholesterol (TC), HDL-C, and low-density lipoprotein-cholesterol (LDL-C) were determined by DxC800 chemistry analyzer (Beckman Coulter, CA, USA). Serum insulin, monocyte chemotactic protein-1 (MCP-1), interleukin-6 (IL-6), tumor necrosis factor-α (TNF-α), leptin, and resistin were measured using MILLIPLEX map kits (Merck Millipore, Darmstadt, Germany).

### Histological examination

Epididymal adipose tissue was embedded in paraffin. Prepared sections were stained with hematoxylin and eosin. Digital images were acquired at ×200 magnification under BX41 light microscopy (Olympus, Tokyo, Japan).

### Real-time quantitative PCR

Total RNA was extracted from epididymal adipose tissue using TRIzol reagent (Thermo Fisher Scientific, MA, USA). RNA concentration was determined using NanoDrop ND-2000 spectrophotometer (Thermo Fisher Scientific, MA, USA). The reverse transcription and amplification reaction were performed with HiScript II Q RT SuperMix for qPCR kit (Vazyme Biotech, Nanjing, China) and AceQ qPCR SYBR Green Master Mix kit (Vazyme Biotech, Nanjing, China), respectively. Real-time quantitative PCR assays were conducted using ABI 7900HT real-time PCR system (Thermo Fisher Scientific, MA, USA). Each sample was measured in triplicate. The primer sequences are listed in Table 1. The relative expression levels were normalized to glyceraldehyde-3-phosphate dehydrogenase and analyzed using the 2^−ΔΔCt^ method.

**Table 1:** Sequences of the primers used in real-time quantitative PCR.

Gene	Forward primer	Reverse primer	Melting temperature (°C)	Product length (bp)	Gen bank code
TLR4	GCATGGCTTACACCACCTCTC	TGTCTCCACAGCCACCAGAT	58	115	NM_021297.3
MyD88	GCTGCTGGCCTTGTTAGACC	CTCGGACTCCTGGTTCTGCT	59	106	NM_010851.3
TRAF6	TTGTCCACACAATGCAAGGAG	TGGCGTCCATGACCTCTTC	55	129	NM_001303273.1
IKKβ	GCAGAAGAGCGAAGTGGACA	AGCCGTTCAGCCAAGACACT	57	111	NM_001159774.1
RIP1	AGAAGAAGCTGCGGTCAGAG	AATTCGTTCCTCCGAGATCC	58	122	NM_001359997.1
IRF3	AGAGGCTTGTGATGGTCAAGG	AGGCTGGCTGTTGGAGATGT	57	119	NM_016849.4
NF-κB p65	CTGGTGCATTCTGACCTTGC	GGTCCATCTCCTTGGTCTGC	56	118	NM_001365067.1
GAPDH	AAGAAGGTGGTGAAGCAGG	GAAGGTGGAAGAGTGGGAGT	59	111	NM_001289726.1

Sequences are listed in the 5′ → 3′ direction.

*TLR4* toll-like receptor 4, *MyD88* myeloid differentiation primary response 88, *TRAF6* tumor necrosis factor receptor-associated factor 6, *IKKβ* inhibitor of nuclear factor kappa B kinase beta, *RIP1* receptor-interacting protein-1, *IRF3* interferon regulate factor 3, *NF-κB p65* nuclear factor-kappa B p65, *GAPDH* glyceraldehyde-3-phosphate dehydrogenase.

### Western blot

Epididymal adipose tissue was homogenized by tissue protein extraction reagent (Thermo Fisher Scientific, MA, USA) and centrifuged at 12,000 × *g* for 15 min at 4 °C. The supernatant protein concentration was measured with BCA protein assay kit (Beyotime Biotechnology, Shanghai, China). Proteins were separated by 10% sodium dodecyl sulfate-polyacrylamide gel electrophoresis and transferred onto a polyvinylidene fluoride membrane (Millipore, MA, USA). After blocking the membrane with 5% nonfat milk at room temperature for 1 h, the membrane was incubated overnight with primary antibodies at 4 °C. Then it was incubated with HRP-conjugated secondary antibodies (Beyotime Biotechnology, Shanghai, China) for 1 h. The signals were detected with ChemiDoc XRS + gel imaging system (Bio-Rad, CA, USA). The densitometric analysis was determined using Image J software. The experiment was repeated three times. The following primary antibodies were used: TLR4 (BS3489, Bioworld, MN, USA), MyD88 (4283S, Cell Signaling Technology, MA, USA), tumor necrosis factor receptor-associated factor 6 (TRAF6, ab33915, Abcam, MA, USA), inhibitor of nuclear factor kappa B kinase beta (IKKβ, ab124957, Abcam, MA, USA), receptor-interacting protein-1 (RIP1, ab202985, Abcam, MA, USA), interferon regulate factor 3 (IRF3, ab68481, Abcam, MA, USA), nuclear factor-kappa B p65 (NF-κB p65, ab32536, Abcam, MA, USA), and glyceraldehyde-3-phosphate dehydrogenase (AG019, Beyotime Biotechnology, Shanghai, China).

### Statistical analysis

Data are represented as mean ± standard deviation. Differences between the means were analyzed by two-tailed one-way analysis of variance followed by Tukey’s post hoc test. *P* < 0.05 was used as the threshold for determining a statistical difference.

## Results

### Effects of FO and PO on glucolipid metabolism, inflammation, and adipokine in C57BL/6J mice

**Fig. 1:**
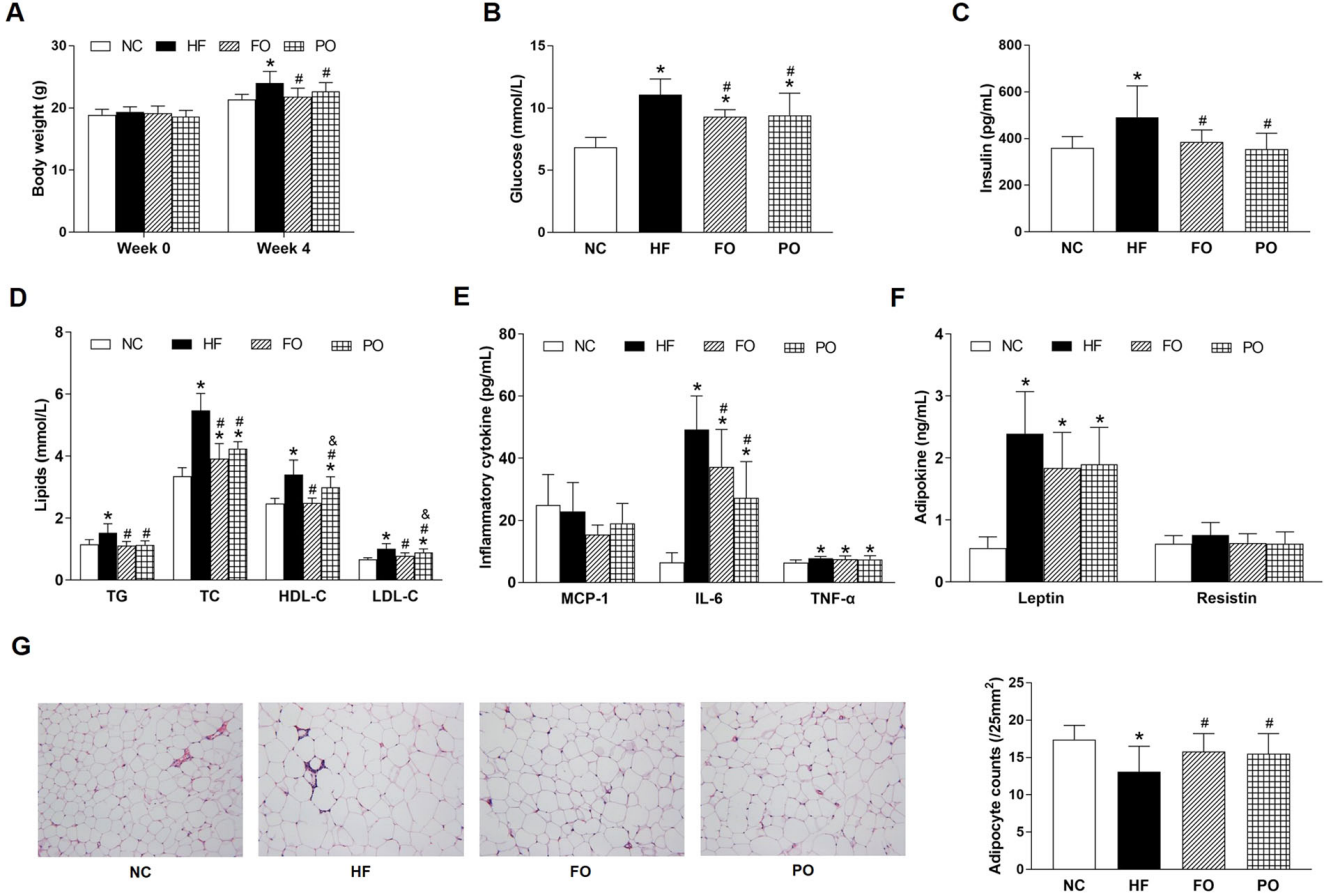
Effects of FO and PO on glucolipid metabolism, inflammation, and adipokine in C57BL/6J mice. **A** Body weight; **B** glucose; **C** insulin; **D** lipids; **E** inflammatory cytokine; **F** adipokine; **G** representative hematoxylin and eosin-stained images (left) and adipocyte counts (right) in adipose tissue. NC normal chow, HF high fat, FO fish oil, PO perilla oil, TG triglyceride, TC total cholesterol, HDL-C high-density lipoprotein cholesterol, LDL-C low-density lipoprotein cholesterol, MCP-1 monocyte chemotactic protein-1, IL-6 interleukin-6, TNF-α tumor necrosis factor-α. Data are presented as the mean ± standard deviation. *P < 0.05, compared with NC; ^#^*P* < 0.05, compared with HF; ^&^*P* < 0.05, compared with FO.

The initial body weight of C57BL/6J mice showed no significant difference among 4 groups (18.9 ± 0.9 g, 19.4 ± 0.8 g, 19.2 ± 1.1 g, and 18.6 ± 1.0 g in NC, HF, FO, and PO, respectively (Fig. 1A). After intervention, body weight, glucose, insulin, TG, TC, HDL-C, LDL-C, IL-6, TNF-α, and leptin were significantly increased in HF than those in NC (Fig. 1A–F). Compared with HF, body weight, glucose, insulin, TG, TC, HDL-C, LDL-C, and IL-6 were significantly decreased in FO and PO (Fig. 1A–E). Hematoxylin and eosin staining showed that FO and PO significantly inhibited HF diet-induced adipocyte hypertrophy by decreasing adipocyte counts (Fig. 1G). HDL-C and LDL-C were significantly higher in PO than those in FO (Fig. 1D). Body weight, glucose, insulin, TG, TC, IL-6, and adipocyte counts had no significant differences between FO and PO (Fig. 1A–E, G). There was no significant difference in body weight before and after FO and PO treatment (Fig. 1A).

### Effects of FO and PO on MyD88-dependent and -independent pathways of TLR4 signaling in C57BL/6J mice

**Fig. 2:**
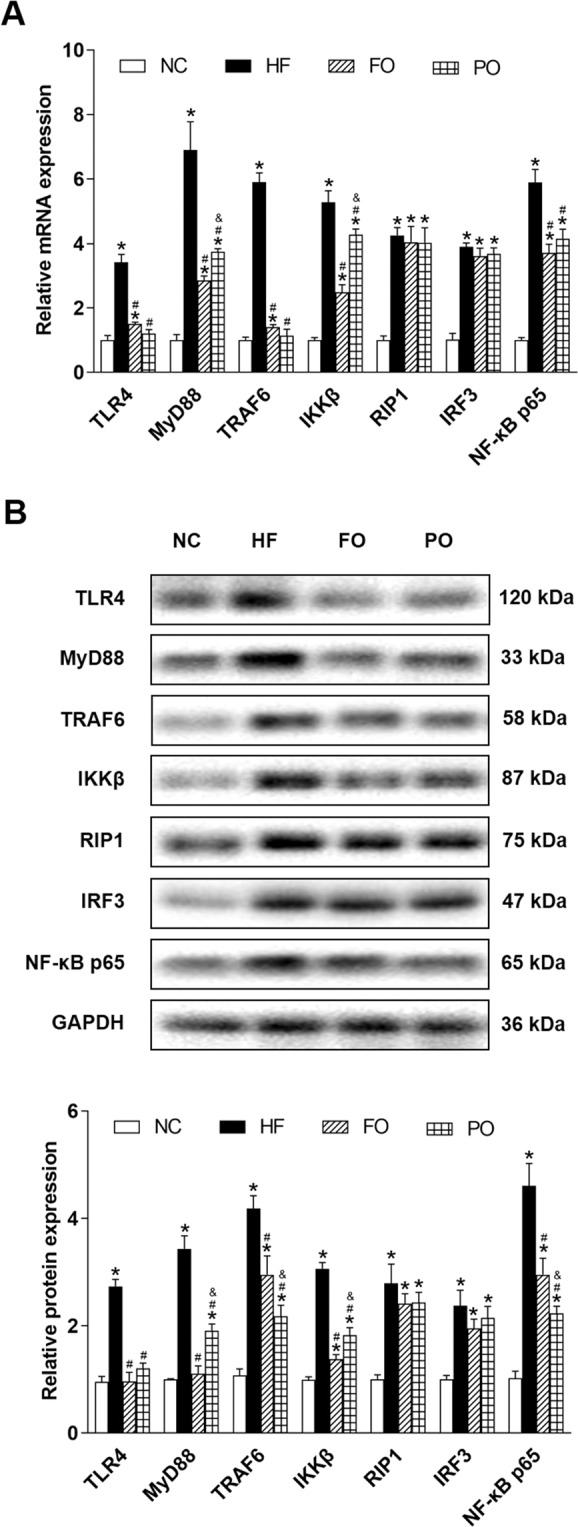
Effects of FO and PO on MyD88-dependent and -independent pathways of TLR4 signaling in C57BL/6J mice. **A** Relative mRNA expression; **B** representative western blots images (upper) and relative protein expression (bottom). NC normal chow, HF high fat, FO fish oil, PO perilla oil, TLR4 toll-like receptor 4, MyD88 myeloid differentiation primary response 88, TRAF6 tumor necrosis factor receptor-associated factor 6, IKKβ inhibitor of nuclear factor kappa B kinase beta, RIP1 receptor-interacting protein-1, IRF3 interferon regulate factor 3, NF-κB p65 nuclear factor-kappa B p65, GAPDH glyceraldehyde-3-phosphate dehydrogenase. Data are presented as the mean ± standard deviation. *P < 0.05, compared with NC; ^#^*P* < 0.05, compared with HF; ^&^*P* < 0.05, compared with FO.

In C57BL/6J mice, both real-time quantitative PCR and western-blot analysis showed that expression of TLR4, MyD88, TRAF6, IKKβ, RIP1, IRF3, and NF-κB p65 were significantly increased in HF than those in NC (Fig. 2A, B). Compared with HF, mRNA and protein expression of TLR4, MyD88, TRAF6, IKKβ, and NF-κB p65 were significantly decreased in FO and PO (Fig. 2A, B). mRNA and protein expression of MyD88 and IKKβ were significantly higher (Fig. 2A, B), whereas protein expression of TRAF6 and NF-κB p65 was significantly lower (Fig. 2B) in PO than those in FO. No statistical differences were observed between these two oils in mRNA and protein expression of TLR4 (Fig. 2A, B), mRNA expression of TRAF6 and NF-κB p65 (Fig. 2A).

### Effects of FO and PO on glucolipid metabolism, inflammation, and adipokine in MyD88−/− mice

**Fig. 3:**
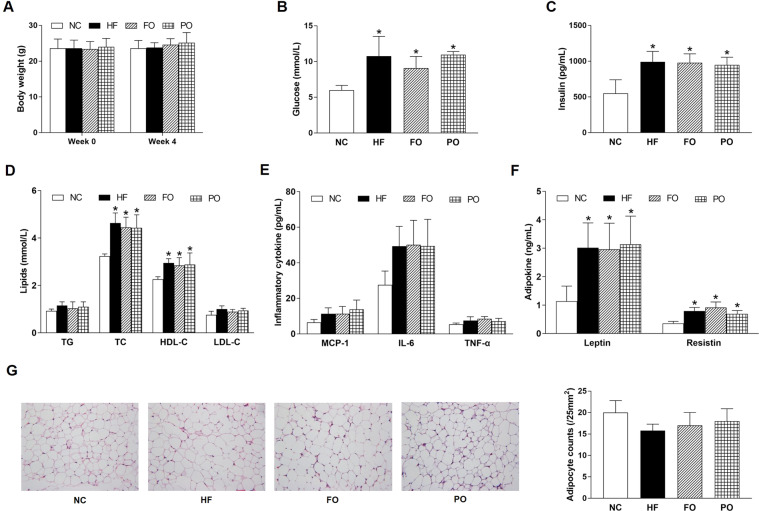
Effects of FO and PO on glucolipid metabolism, inflammation, and adipokine in MyD88−/− mice. **A** Body weight; **B** glucose; **C** insulin; **D** lipids; **E** inflammatory cytokine; **F** adipokine; **G** representative hematoxylin and eosin-stained images (left) and adipocyte counts (right) in adipose tissue. NC normal chow, HF high fat, FO fish oil, PO perilla oil, TG triglyceride, TC total cholesterol, HDL-C high-density lipoprotein cholesterol, LDL-C low-density lipoprotein cholesterol, MCP-1 monocyte chemotactic protein-1, IL-6 interleukin-6, TNF-α tumor necrosis factor-α. Data are presented as the mean ± standard deviation. *P < 0.05, compared with NC; ^#^*P* < 0.05, compared with HF; ^&^*P* < 0.05, compared with FO.

The initial body weight of MyD88−/− mice showed no significant difference among 4 groups (23.6 ± 2.6 g, 23.6 ± 2.3 g, 23.4 ± 2.1 g, and 24.0 ± 2.4 g in NC, HF, FO, and PO, respectively (Fig. 3A). After intervention, glucose, insulin, TC, HDL-C, leptin, and resistin were significantly increased in HF than those in NC (Fig. 3B–D, F). There was a trend toward increased TG, LDL-C, MCP-1, IL-6, TNF-α (Fig. 3D, E), and decreased adipocyte counts (Fig. 3G) in HF compared with NC, although the differences did not reach significance. Glucose, insulin, TC, HDL-C, leptin, and resistin in FO and PO had no statistical differences from HF (Fig. 3B–D, F). There was no significant difference in body weight before and after FO and PO treatment (Fig. 3A).

### Effects of FO and PO on MyD88-independent pathway of TLR4 signaling in MyD88−/− mice

**Fig. 4:**
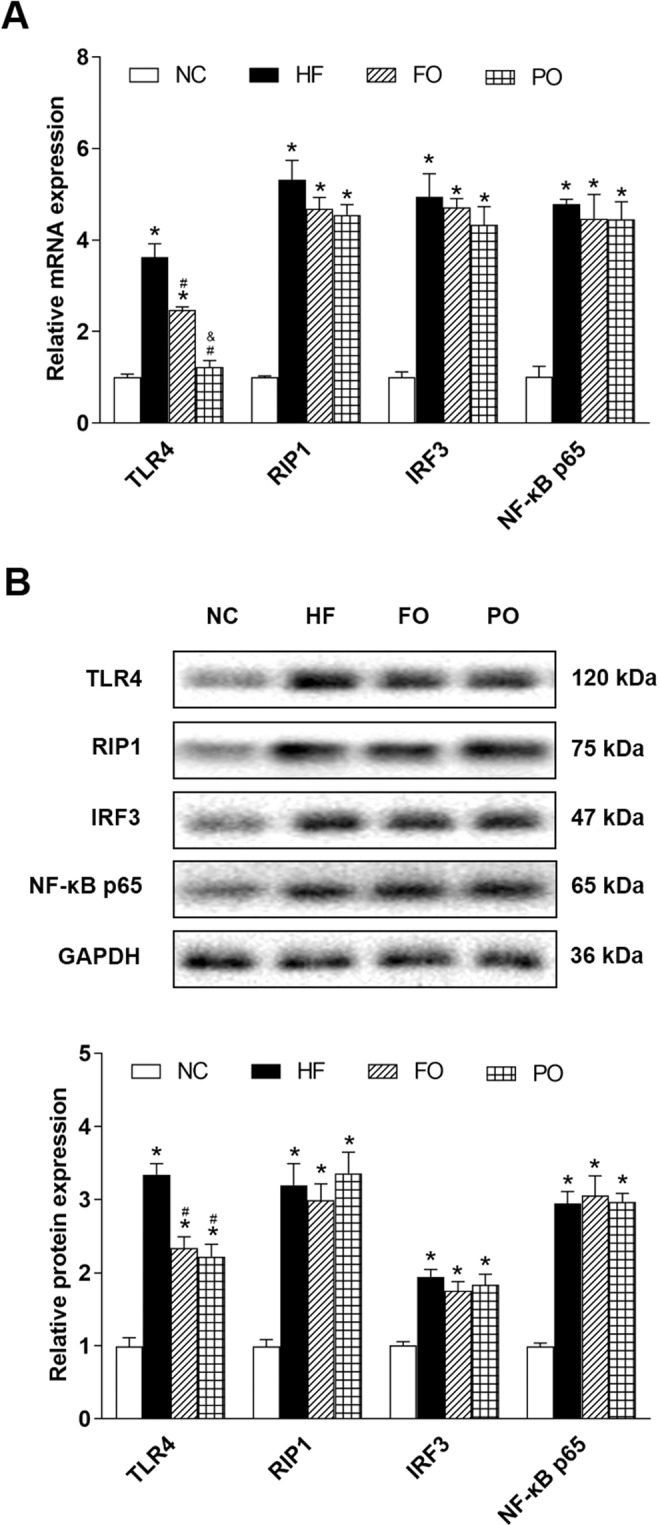
Effects of FO and PO on MyD88-independent pathway of TLR4 signaling in MyD88−/− mice. **A** Relative mRNA expression; **B** representative western blots images (upper) and relative protein expression (bottom). NC normal chow, HF high fat, FO fish oil, PO perilla oil, TLR4 toll-like receptor 4, RIP1 receptor-interacting protein-1, IRF3 interferon regulate factor 3, NF-κB p65 nuclear factor-kappa B p65, GAPDH glyceraldehyde-3-phosphate dehydrogenase. Data are presented as the mean ± standard deviation. *P < 0.05, compared with NC; ^#^*P* < 0.05, compared with HF; ^&^*P* < 0.05, compared with FO.

In MyD88−/− mice, both real-time quantitative PCR and western-blot analysis showed that expression of TLR4, RIP1, IRF3, and NF-κB p65 were significantly increased in HF than those in NC (Fig. 4A, B). Compared with HF, FO, and PO produced significant reduction of TLR4, but had no significant effects on RIP1, IRF3, and NF-κB p65 (Fig. 4A, B). mRNA expression of TLR4 was significantly lower in PO than that in FO (Fig. 4A). No statistical difference was observed between these two oils in protein expression of TLR4 (Fig. 4B).

## Discussion

In the present study, we observed that FO and PO had similar adjusting roles on body weight, glucose, insulin, TG, TC, IL-6, and adipocyte counts in C57BL/6J mice fed a HF diet, indicating comparable regulations in metabolic disorders and inflammation between marine and plant ω−3 PUFA. In line with our observations, it was shown previously that both FO and PO attenuated HF diet-induced hypercholesterolemia in Sprague Dawley rats^17^. Likewise, the effect of flaxseed oil in reducing insulin was similar to FO in diabetic patients with coronary heart disease^18^. The mRNA expression of IL-6 was downregulated by FO and flaxseed oil in kidney of streptozotocin-nicotinamide-induced Wistar rats^19^.

There are, however, also examples of inconsistent results. FO, but not flaxseed oil, decreased TG in hypercholesterolemic adults^20^. FO was more potent than flaxseed oil to ameliorate trimethylamine-N-oxide-induced atherogenesis by lowering TC in ApoE−/− mice^21^. On the other hand, plant ω−3 PUFA has superiority to marine ω−3 PUFA. Camelina sativa oil, rather than fatty fish or lean fish, decreased TC in subjects with impaired glucose metabolism^22^. Safflower oil-rich diet with flaxseed oil presented lower glucose after insulin injection than that with sardine oil or tuna oil in ob/ob mice^23^. Compared with EPA oil and DHA oil, ALA-rich chia oil improved glucose tolerance in Wistar rats fed a high-carbohydrate and HF diet^24^.

These inconsistencies may be partly explained by differences in oil type, host species, and illness status. In addition, the ratio of ω−3 to ω−6 PUFA has implications for metabolic health. Despite the similar ω−3 PUFA content, butter with high ratio of ω−3 to ω−6 PUFA displayed lower glucose, insulin resistance, and TC than that with low ratio of ω−3 to ω−6 PUFA in C57BL/6 mice^25^. Our research group previously showed that low linoleic acid/ALA ratio provided cardiovascular benefits at low PUFA concentration in EA.hy926 cell line^26^. More direct comparative studies are required to evaluate the differential effects of ω−3 PUFA from various sources on metabolic dysfunction.

HDL-C is usually considered a protective factor against coronary heart disease. Intriguingly, HF diet feeding increased HDL-C, which was fully reversed by FO and partly reversed by PO in C57BL/6J mice. Likewise, OLETF rats fed FO had lower HDL-C than those fed PO^27^. On both the low and high saturated fatty acids diets, the presence of FO decreased HDL-C in healthy men^28^. Cholesteryl ester transfer protein gene variants were strongly associated with high HDL-C but had no significant association with cardiovascular risk^29^. These findings suggested that besides the quantity, the quality of HDL-C may also be a vital indicator. In fact, the function of HDL-C varies depending on HDL subfractions, which can be further distinguished by size, shape, and density^30–32^. Therefore, in-depth analysis of HDL profile bears a potential for the improved assessment of metabolic impairments.

A chronic, low-grade inflammation in adipose tissue is essential to the development of obesity. Our results indicated that MyD88-dependent and -independent pathways of TLR4 signaling were activated in adipose tissue of HF diet-fed C57BL/6J mice. Consistently, individuals with obese and overweight showed increased mRNA expression of TLR4 and MyD88 in adipose tissue^33^. HF diet feeding increased mRNA expression of NF-κB in adipose tissue of C57BL/6J mice^34^. In contrast, FO and PO inhibited MyD88-dependent instead of -independent pathway of TLR4 signaling. Similarly, HF diet-fed C57BL/6J mice that received DHA isolated from FO exhibited decreased mRNA expression of NF-κΒ in adipose tissue^35^. A functional blended oil composed of a high level of ALA inhibited mRNA expression of TLR4 and NF-κB in adipose tissue of obese Wistar rats^36^.

To further verify the inhibitory effects of FO and PO on MyD88-dependent pathway of TLR4 signaling, MyD88 gene knockout was performed in C57BL/6J mice. We identified that metabolism abnormalities and inflammation still appeared in HF, but the protective effects of FO and PO were abolished. Moreover, FO and PO had no impacts on mRNA and protein expression of MyD88-independent pathway of TLR4 signaling, indicating the beneficial effects of FO and PO were dependent on MyD88. In accordance with our findings, MyD88−/− mice fed a HF diet presented increased glucose, TC, and leptin^37^. Contrary, MyD88−/− mice fed a FO-enriched HF diet for 11 weeks gained less body weight and had smaller adipocyte size than those fed a lard-enriched HF diet^15^. Such discrepancy may be due to our relatively short administration time and different control. Exact mechanism underlying these effects warrants further exploration.

The main limitation of our study is that the dosage regimen of EPA + DHA and ALA is differ, which leads to difficulties in characterizing the efficacy of ω−3 PUFA from different sources. In reality, it is hard to apply purified EPA, DHA, and ALA in animal experiments owing to their high costs. Alternatively, we used EPA + DHA-rich FO and ALA-rich PO at the same amount. In addition, only a single oil dose and time point were administrated, so the information on dose-effect and time-effect has yet to be described. Further functional and fundamental researches are needed to progress in the health benefits of ω−3 PUFA.

In summary, the present study demonstrates that FO and PO have the similar protective effects on metabolic disorders and inflammation through inhibiting TLR4 signaling in a manner dependent on MyD88. These findings highlight plant ω−3 PUFA as an attractive alternative source of marine ω−3 PUFA and reveal a mechanistic insight for preventive benefits of ω−3 PUFA in obesity and related metabolic diseases.

## Supplementary information

Table S1-S3
