# Supplementary material for: MyD88 determines the protective effects of fish oil and perilla oil against metabolic disorders and inflammation in adipose tissue from mice fed a high-fat diet
Source: Nutr Diabetes. 2021 Jun 17;11:23. doi: 10.1038/s41387-021-00159-y (PMC8225863; doi:10.1038/s41387-021-00159-y)
Supplement: Supplementary file 1 — Table S1-S3 [file 41387_2021_159_MOESM1_ESM.docx]

Table S1. The fatty acid composition of the diets (Percentage, %)

| Fatty acid | AIN-93M | D12451 |
| --- | --- | --- |
| C16:0 | 11.1 | 13.0 |
| C18:0 | 3.8 | 5.3 |
| C16:1 n-7 | 1.5 | 1.6 |
| C18:1 n-9 | 22.4 | 25.3 |
| C18:2 n-6 | 51.7 | 46.8 |
| C18:3 n-3 | 6.7 | 5.9 |

Only fatty acids detected in > 1% of total fatty acids are shown.

Table S2. The fatty acid composition of fish oil

| Fatty acid | Percentage (%) |
| --- | --- |
| C20:4 n-6 | 2.3 |
| C20:5 n-3 | 38.3 |
| C22:1 | 4.6 |
| C23:0 | 2.1 |
| C22:5 n-6 | 1.6 |
| C22:4 n-6 | 2.8 |
| C22:6 n-3 | 48.1 |

Only fatty acids detected in > 1% of total fatty acids are shown.

Table S3. The fatty acid composition of perilla oil

| Fatty acid | Percentage (%) |
| --- | --- |
| C16:0 | 4.6 |
| C18:0 | 2.3 |
| C18:1 n-9 | 11.7 |
| C18:2 n-6 | 12.0 |
| C18:3 n-3 | 68.4 |

Only fatty acids detected in > 1% of total fatty acids are shown.
